# Successful Treatment of Colorectal Anastomotic Stricture by Using Sphincterotomes

**DOI:** 10.3389/fsurg.2014.00022

**Published:** 2014-06-20

**Authors:** Tzu-An Chen, Wei-Lun Hsu

**Affiliations:** ^1^Division of Colorectal Surgery, Department of Surgery, Taiwan Landseed Hospital, Taoyuan, Taiwan; ^2^Division of Gastroenterology, Department of Internal Medicine, Taiwan Landseed Hospital, Taoyuan, Taiwan

**Keywords:** colorectal anastomosis, stricture, sphincterotomes, colorectal surgery, colonoscopy

## Abstract

Colorectal or colocolic anastomotic stricture is a common complication after colorectal surgery. Traditionally, endoscopic balloon dilation technique was used for those patients with symptomatic stricture. The use of electroincision (radial incisions of the scar) along with pneumatic balloon dilation was reported with good result in literature. We present a novel method for relieving colorectal anastomotic stricture by using sphincterotomes, which is indicated for use in the cannulation of the biliary ducts and the transendoscopic sphincterotomy of the papilla of Vater and the sphincter of Oddi. The use of sphincterotomes in upper GI tract anastomotic stricture was reported before, but the experience in managing lower GI tract was pending. Based on our preliminary report, sphincterotomes can be an effective and safe treatment option for colorectal anastomotic stricture.

## Introduction

Anastomotic stricture is a relatively common complication of low anterior resection that requires further management if an obstruction occurs (1). Direct digital dilatation or transrectal surgical treatment is possible if the anastomosis is located in the lower rectum. Endoscopic balloon dilatation is the most effective method for any patients on whom these lower rectum procedures cannot be performed (2–4), although repeated dilation over time is often required to achieve normal bowel function (5). Dilation can be combined with other procedures such as neodymium: yttrium–aluminum–garnet laser treatment (6), electroincision (7), and electrocautery (8). We present a novel method for relieving anastomotic stricture by using sphincterotomes, which is indicated for use in the cannulation of the biliary ducts and the transendoscopic sphincterotomy of the papilla of Vater and the sphincter of Oddi. Our preliminary report revealed that our method was easily and safely performed and the long-term result seems good.

## Case Series

### Case 1

A 47-year-old male patient received multiple colonoscopic reductions for sigmoid volvulus. Because of the recurrent episodes, the patient finally accepted surgery. After resection of the redundant sigmoid colon, an end-to-side colorectal anastomosis was completed with a double-stapled technique. He was later discharged in good condition with fairly regular bowel movements.

**Figure 1:**
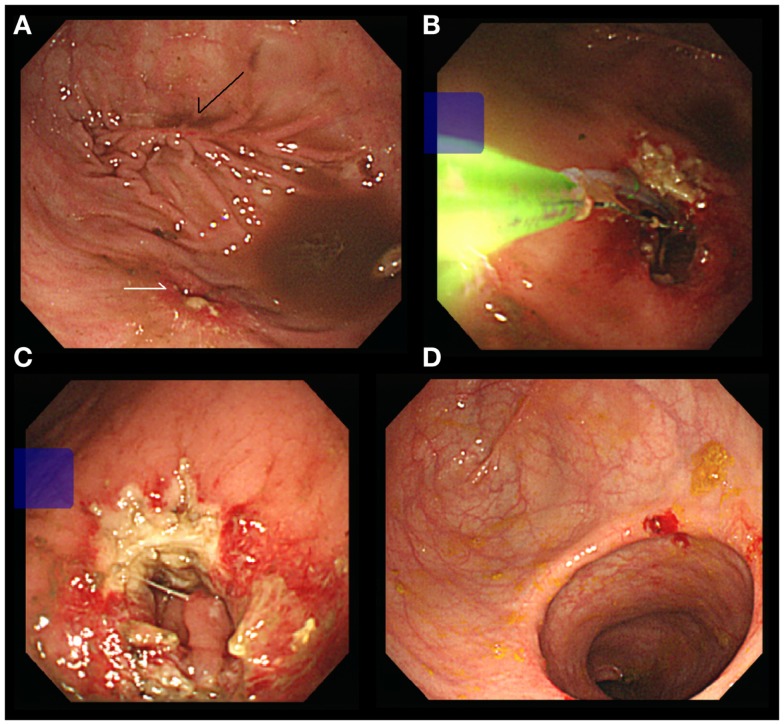
**(A)** Stricture of anastomosis (white arrow). Note the previous stapled linear scar (black arrow). **(B)** Using Sphincterotomes to release the circumferential scar. **(C)** Complete the bilateral incisions. **(D)** Increased the diameter of anastomosis.

One month after discharge, the patient was hospitalized again because of abdominal pain and 7 days of constipation. Diffuse bowel dilation was noted in the X-ray study. A colonoscopy exam was performed and a stricture of colorectal anastomosis was identified, where the colonoscope was unable to pass through the anastomosis (Figure 1A). We changed the instrument to a panendoscope for using sphincterotomes (Clevercut; Olympus, Tokyo, Japan) to release the circumferential scar of the anastomosis (Figure 1B). The other incisions were made in different directions (Figure 1C). After the procedure, a massive stool passage was noted and the obstructive symptom was relieved. Four months later, we performed another colonoscopy for surveillance. The diameter of the anastomosis had increased obviously and the underlying mucosa was growing well (Figure 1D). No more symptoms of intestinal obstruction were noted after 18-month follow up.

### Case 2

**Figure 2:**
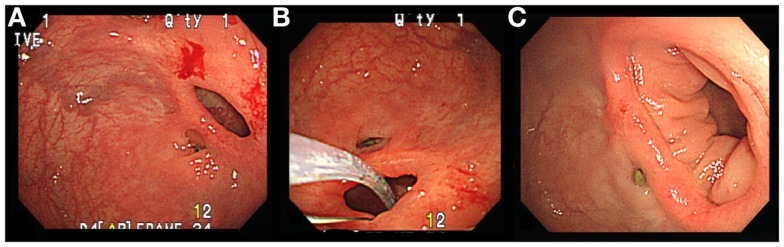
**(A)** Stricture of anastomosis. **(B)** Using Sphincterotomes to release the circumferential scar. **(C)** Increased the diameter of anastomosis.

A 73-year-old male patient received low anterior resection with a double-stapled technique for rectosigmoid colon cancer. The operation and hospital course were uneventful. Constipation and abdominal fullness were complained later. The surveillance colonoscopy was performed 6 months after operation. Stricture of the anastomosis was noted and we used sphincterotomes for releasing the circumferential scar again (Figures 2A,B). The obstructive symptoms were relieved after the procedure and the lumen was markedly increased in the following-up exam (Figure 2C).

## Discussion

Although circular stapling anastomosis of the rectum has been used widely and has been regarded as a safe and quick technique, the development of frequent anastomotic strictures is the major postoperative complication of this procedure (9, 10). The incidence of colorectal anastomotic strictures varies from 3 to 30%, and is considered to be related to factors including radiation (11) and anastomotic leakage (12). Most of these anastomotic strictures are simple constrictions that can be successfully treated using dilation or endoscopic alternatives. However, up to 28% of patients require a surgical correction. This can be technically difficult, and a permanent colostomy is possible (13).

**Figure 3:**
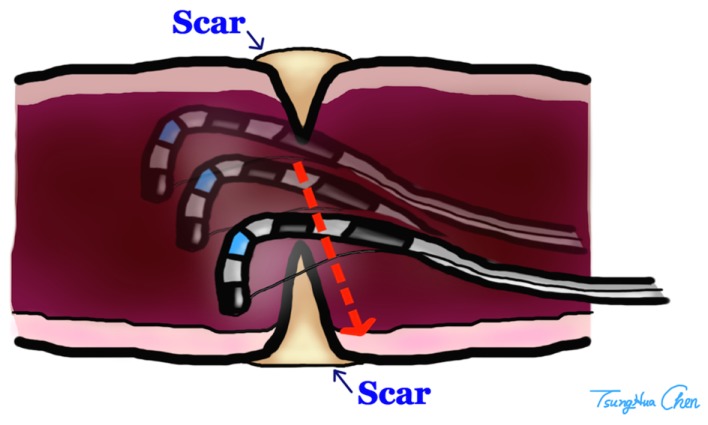
**The depth of incision was limited by the arms of Sphincterotomes**.

Endoscopic incision for the treatment of anastomotic stenosis in the upper gastrointestinal tract is well accepted (14), but its application for colorectal anastomotic strictures is limited. The sphincterotome is a bow-like device, which has an electrosurgical cutting wire at the distal end of the catheter. A monopolar power source is connected to the catheter at an electrode connector on the handle. During a sphincterotomy activation of the power source causes electrical current to pass along an insulated portion of the wire within the catheter to the exposed cutting wire. A retractable plunger on the control handle permits flexing of the catheter tip upward by pulling on the cutting wire. This flexing assists with aligning the cutting wire and maintaining contact of the wire with the scarred anastomosis while the catheter is pulled back, incising the circular scar of anastomosis by electrocauterization. Because of the bilateral plastic limbs of the sphincterotome, the depth of electrocauterization was limited and perforation of bowel wall could be avoided (Figure 3). We made three or four incisions at different directions to release the stricture. The purpose of the incision was breakdown of the membranous circular scar, and we preferred multiple shallow incisions but not one deep incision with curative intent. We believe that if the strength of stricture was released by multiple incisions, the lumen would be dilated by the following stool passage. One curative deep incision was not necessary so the risk of bowel injury could be diminished.

Some authors also reported small case series about using endoscopic incisions plus balloon dilation for the anastomotic stricture (15, 16). Based on our result, additional balloon dilation might not be necessary if the multiple incisions were completed and the membranous scar was demolished. Recently, some authors tried to treat benign colorectal strictures by stenting (17). Although the use of self-expanding metal stents to treat obstruction colorectal tumors has been commonly described in the literature, the application of stenting to benign stricture is uncommon. The long-term reliability of stenting is questioned; migration, erosion, pressure necrosis, and bleeding all have been reported (18). In our opinion, endoscopic self-expanding metal stent placement as a bridge to surgery is an option for acute malignant colonic obstruction. For the long-term usage in benign anastomotic stricture, colonic stenting is not encouraged.

The application of sphincteromes seems suitable for colorectal anastomotic strictures, which was easily performed, and yielded an effective result. The present outcome was also satisfactory. However, the risk and the long-term results of this method could not be assessed because of limited experiences.

## Authors Contribution

We are accepting full responsibility for the conduct of the study. Specific author contributions: Tzu-An Chen looked after the patients and wrote the report. Tzu-An Chen and Wei-Lun Hsu completed the procedures.

## Conflict of Interest Statement

The authors declare that the research was conducted in the absence of any commercial or financial relationships that could be construed as a potential conflict of interest.
